# Machining feature recognition based on deep neural networks to support tight integration with 3D CAD systems

**DOI:** 10.1038/s41598-021-01313-3

**Published:** 2021-11-12

**Authors:** Changmo Yeo, Byung Chul Kim, Sanguk Cheon, Jinwon Lee, Duhwan Mun

**Affiliations:** 1 School of Mechanical Engineering, Korea University, 145, Anam-ro, Seongbuk-gu, Seoul, 02841 South Korea; 2 School of Mechanical Engineering, Korea University of Technology and Education, 1600 Chungjeol-ro, Byeongcheon-myeon, Dongnam-gu, Cheonan-si, Chungcheongnam-do 31253 South Korea; 3 Department of Integrative Systems Engineering, Ajou University, 206, Worldcup-ro, Yeongtong-gu, Suwon, 16499 South Korea

**Keywords:** Aerospace engineering, Mechanical engineering

## Abstract

Recently, studies applying deep learning technology to recognize the machining feature of three-dimensional (3D) computer-aided design (CAD) models are increasing. Since the direct utilization of boundary representation (B-rep) models as input data for neural networks in terms of data structure is difficult, B-rep models are generally converted into a voxel, mesh, or point cloud model and used as inputs for neural networks for the application of 3D models to deep learning. However, the model’s resolution decreases during the format conversion of 3D models, causing the loss of some features or difficulties in identifying areas of the converted model corresponding to a specific face of the B-rep model. To solve these problems, this study proposes a method enabling tight integration of a 3D CAD system with a deep neural network using feature descriptors as inputs to neural networks for recognizing machining features. Feature descriptor denotes an explicit representation of the main property items of a face. We constructed 2236 data to train and evaluate the deep neural network. Of these, 1430 were used for training the deep neural network, and 358 were used for validation. And 448 were used to evaluate the performance of the trained deep neural network. In addition, we conducted an experiment to recognize a total of 17 types (16 types of machining features and a non-feature) from the B-rep model, and the types for all 75 test cases were successfully recognized.

## Introduction

**Figure 1:**
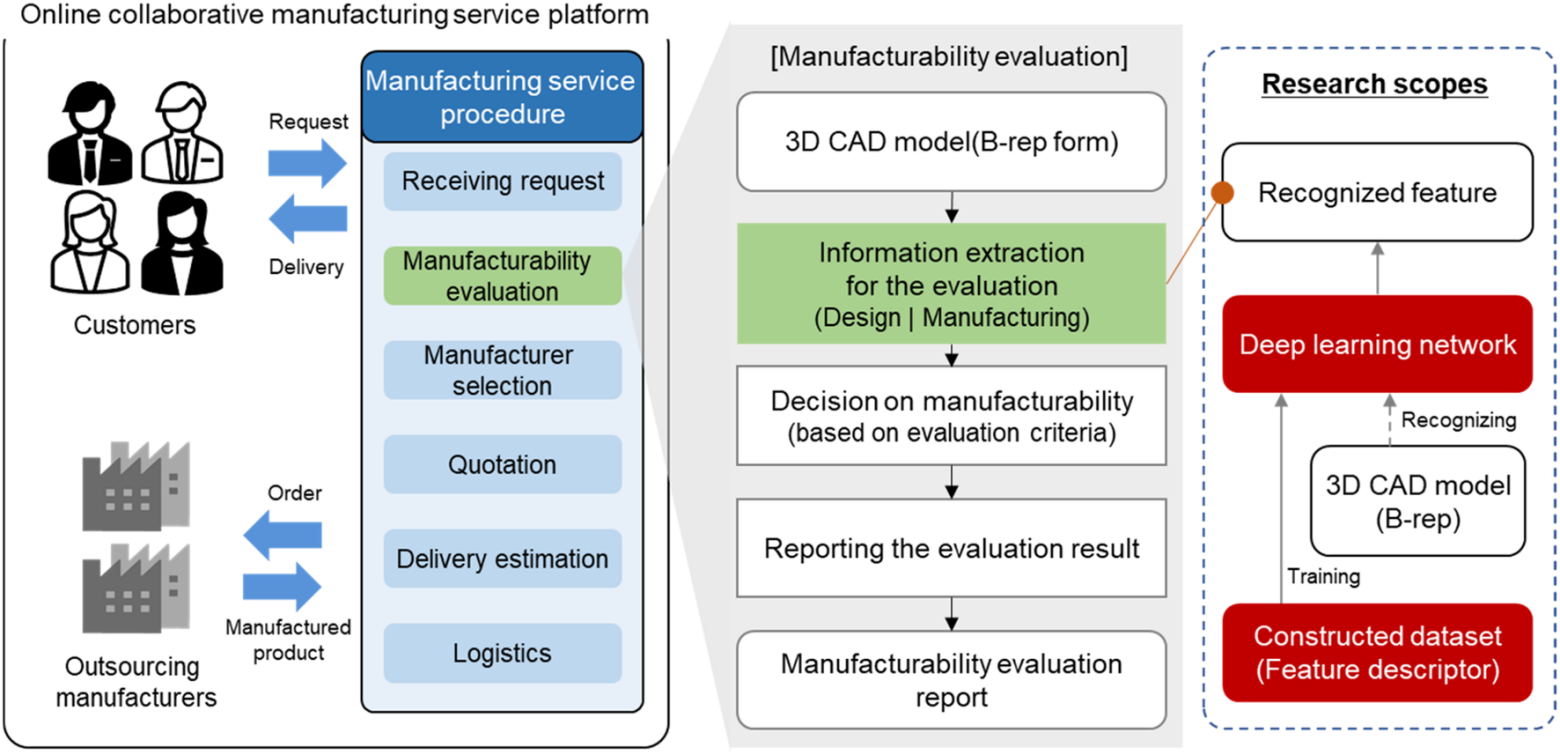
Research motivation.

Due to structural changes in the manufacturing industry following the development of information technology, the demand for online manufacturing support has increased worldwide. Following this trend, the online manufacturing support platform industry is growing. Online manufacturing support, as shown in Fig. 1, is an intermediary service that evaluates the manufacturability of three-dimensional (3D) computer-aided design (CAD) models uploaded online by customers (individuals or businesses) and selects manufacturers. Furthermore, it calculates estimates, requests for production to manufacturers, and manages the entire process of delivering products to customers^1^. Manufacturability evaluation means the process of determining manufacturing methods, level of difficulty in manufacturing, manufacturability, and manufacturing costs for a customer-supplied CAD model^2^. Automation of the manufacturability evaluation process is essential for providing smooth online manufacturing support services. This is because it minimizes human interference and enables quick response regarding numerous manufacturing requests.

For automated manufacturability evaluation, machining features, which are necessary for making the designed shape from stock, are essential. Machining features include machining tools and processing parameters to be used to manufacture products. Using these, the manufacturing errors and manufacturing time can be identified through machining simulation and the necessary manufacturing tools, materials, and time can be ultimately estimated. However, as designers and manufacturers are different in the intermediary service platform, machining feature information is not provided for product security reasons. Therefore, for manufacturability evaluation, we have no choice but to use boundary representation (B-rep) models that contain only product shape information, not including feature information. So, it is necessary to find machining features from the B-rep model, which is possible through machining feature recognition.

Common methods for recognizing the machining features of machine processing are based on algorithms including graph-based, volume decomposition, and hint-based, and similarity-based methods. For algorithm-based methods, however, the recognition rate for complicated shapes is low. Moreover, it is difficult to recognize when machining features intersect with each other, even if the shapes are not complicated. In addition, most algorithms have high complexity and long recognition time^3–16^. Recently, studies on the recognition of machining features using deep learning to compensate for the shortcomings of algorithm-based methods have been reported. When using deep learning, data should typically be represented in a lattice-structured form. Therefore, since the B-rep model cannot be used as it is, previous studies converted 3D shapes into voxel, projected images, point clouds, etc., for using the deep learning^17–26^.

However, in the course of the conversion, the following problems arise. First, the geometric information of the model may be significantly lost or changed. For example, local features with a size smaller than the resolution of the converted model are lost. Moreover, in the case of formats other than mesh, accurate representation of a curved surface is difficult. Second, the association between the information obtained through deep learning and topology elements of the original B-rep model is difficult to identify. When the areas where features exist (a set of voxels) are detected through deep learning, the corresponding set of faces of the B-rep model should be found. However, due to the loss or change of the model's geometric information during the conversion, finding a set of faces corresponding to the detected area is difficult. This problem can be seen as a highly studied problem of persistent naming in the CAD field^27,28^. The above problem makes it difficult to integrate information obtained from deep learning and 3D CAD systems that have to deal with B-rep models when realizing online manufacturing support platforms.

To solve these problems, in this study, we propose a method to ensure tight integration^29^ with 3D CAD systems. The proposed method defines a feature descriptor for each face of the shape. It defines and trains deep neural network models that use feature descriptors as input and feature types as outputs, and then it uses them to recognize machining features to which each face belongs. Since the proposed method recognizes machining features for each face, the recognition results of deep neural networks can be directly related to the original B-rep model.

This study has the following academic contributions. First, we propose a deep learning-based machining feature recognition architecture that enables tight integration with 3D CAD systems. Second, we propose the concept and data structure of the feature descriptor and utilize it in the feature recognition process. Third, we develop a deep neural network that classifies machining feature types by receiving the input of the feature descriptor. To the best of our knowledge, this study is the first to recognize machining features using deep learning to enable integration with 3D CAD systems.

The structure of this study is as follows. Section “Related studies” presents the related research. Section “System construction and process” proposes a deep learning-based machining feature recognition method for tight integration with 3D CAD systems. Section “Machining feature recognition using deep learning technology” explains the recognition of machining features based on deep neural networks, a key element of the proposed architecture. Section “Implementation and experimentation” discusses the implementation and experimental results. Finally, the conclusions and future research directions are presented in “Conclusions”.

## Related studies

Representative methods for recognizing machining features in B-rep type 3D CAD models include graph-based, convex decomposition, cell-based decomposition, hint-based, similarity-based, and artificial neural-network-based methods.

The graph-based method represents the adjacent relation between the faces and edges of the whole shape and features as a graph structure and then finds and recognizes sub-graphs of the features in the graph of the whole shape. Joshi and Chang^3^ used heuristics to solve feature intersection problems in machining feature recognition. Chuang and Henderson^4^ utilized the graph-based method using vertex and edge to search for patterns in machining features. Gavankar and Henderson^5^ proposed a method for separating the connections after showing that the graph connection between the protrusion and depression areas is doubly constructed. Graph-based methods have the advantage of being able to easily add new features to be recognized and apply to various domains. However, it is difficult to apply when the topology structure of features is variable or features intersect. In addition, since graph search takes exponential time, it is difficult to apply to complicated shapes.

In convex decomposition and cell-based decomposition methods, features are recognized from simple shapes after complicated shapes are decomposed into simple ones. The convex decomposition method decomposes the target shape using the convex hull and delta volume. Tang and Woo^6^ proposed alternating sum of volumes (ASV), which allows features to be recognized by decomposing shapes in the convex decomposition method. However, there is a problem in ASV when decomposition on a particular shape does not converge. Kim^7^ proposed alternating sum of volumes with partitioning (ASVP) decomposition to solve ASP problems and used it to recognize features. The convex decomposition method can well recognize features even when they intersect, but since it cannot be applied to shapes with curved surfaces, fillets or rounds need to be removed and curved parts need to be converted to polyhedron in advance.

In cell-based decomposition, the shapes are decomposed into simple cells and the decomposed cells are recombined to form a maximum volume to find features. Sakurai and Dave^8^ proposed a method of decomposing the shapes into small cells with simple shapes and recombining these cells to form large volumes. Woo^9^ presented a method to perform cell-based decomposition faster than traditional cell-based decomposition. Cell-based decomposition methods can also be applied even when features intersect, and feature recognition is possible when secondary curved surfaces are included. However, it cannot be applied to complicated shapes because the process of recombining cells has high time complexity.

The hint-based method starts with minimal traces or hints for feature recognition, instead of finding complete feature patterns, and finds features through a geometric inference process for surrounding shapes. Vandenbrande and Requisha^10^ developed an object-oriented feature finder (OOFF), an algorithm that explores hints from faces regarding slots, holes, and pockets. Regli^11^ developed an algorithm to explore hints using edges and vertices rather than faces. Han and Requicha^12^ developed incremental feature finder (IF^2^), which extends the functionality of OOFF. Disadvantageously, recognition rules need to be individually defined for each feature in hint-based methods.

The similarity-based method recognizes features by examining how similar two comparison shapes are. Hong et al.^13^ generated low- and high-resolution models from the B-rep model via multi-resolution modeling. They used the low-resolution model for comparing the whole shape, and the high-resolution model for comparing detailed shapes. Ohbuchi and Furuya^14^ and Liu et al.^15^ proposed a method to compare the similarity of the shapes contained in images after generating images of the 3D model from multiple viewpoints. Sánchez-Cruz and Bribiesca^16^ compared similarities after converting 3D models to voxel formats. However, this method cannot consider the properties of faces or other properties that features have, such as adjacency relations.

Recently, methods have been proposed for recognizing features in 3D models using artificial neural networks^17–21^. Jian et al.^22^ proposed an improved novel bat algorithm (NBA) incorporating NBA, which was developed to complement existing neural networks with long learning time, using a graph-based method. Zhang et al.^23^ recognized 24 kinds of machining features by applying 3D convolutional neural networks. Shi et al.^24^ proposed MsvNet, a deep learning technology based on multiple sectional view (MSV) representation, which was used to recognize machining features. Peddireddy et al.^25^ proposed a method to identify machining processes based on 3D convolutional neural networks and transfer learning. Zhang et al.^26^ constructed the PointwiseNet based on 3D point clouds and showed high performance by applying the constructed deep learning model to 3D shape retrieval. However, in the artificial neural network-based method, tightly integrating the B-rep model with 3D CAD systems is difficult because the B-rep model cannot be directly used and it needs to be converted to other formats, such as a voxel. In particular, the accurate identification of the faces on the B-rep model is difficult, which corresponds to the detection of areas corresponding to features from a voxel.

## System construction and process

**Figure 2:**
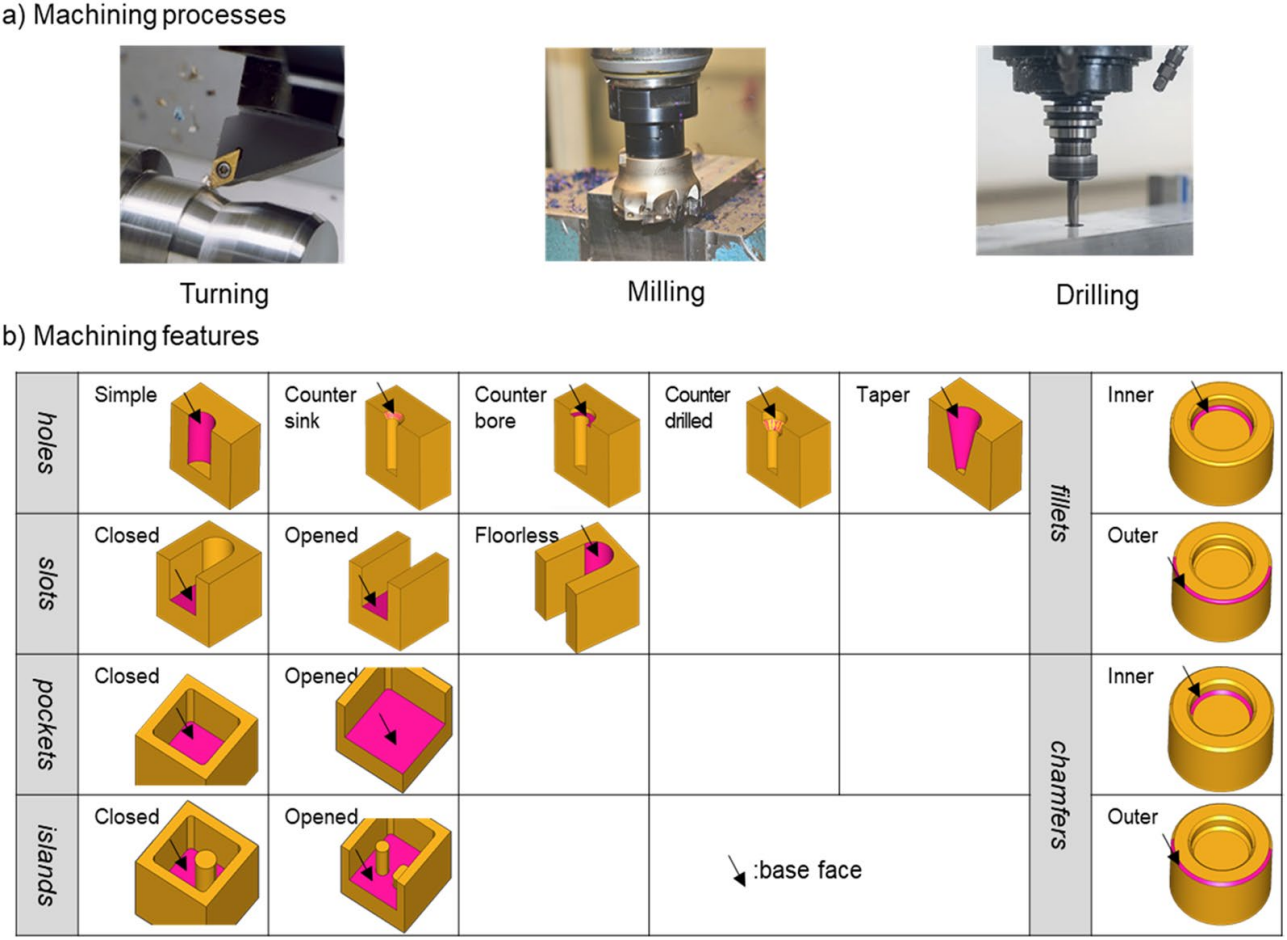
Machining feature types to be recognized from a B-rep model.

In this study, the target range of machining methods is limited to tuning, milling, and drilling as shown in Fig. 2a. Sixteen machining features of Fig. 2b are to be recognized. The machining features to recognize are classified as five features of hole-related type, three features of slot-related type, two features of pocket-related type, two features of island-related type, two features of fillet-related type, and two features of chamfer-related type.

**Figure 3:**
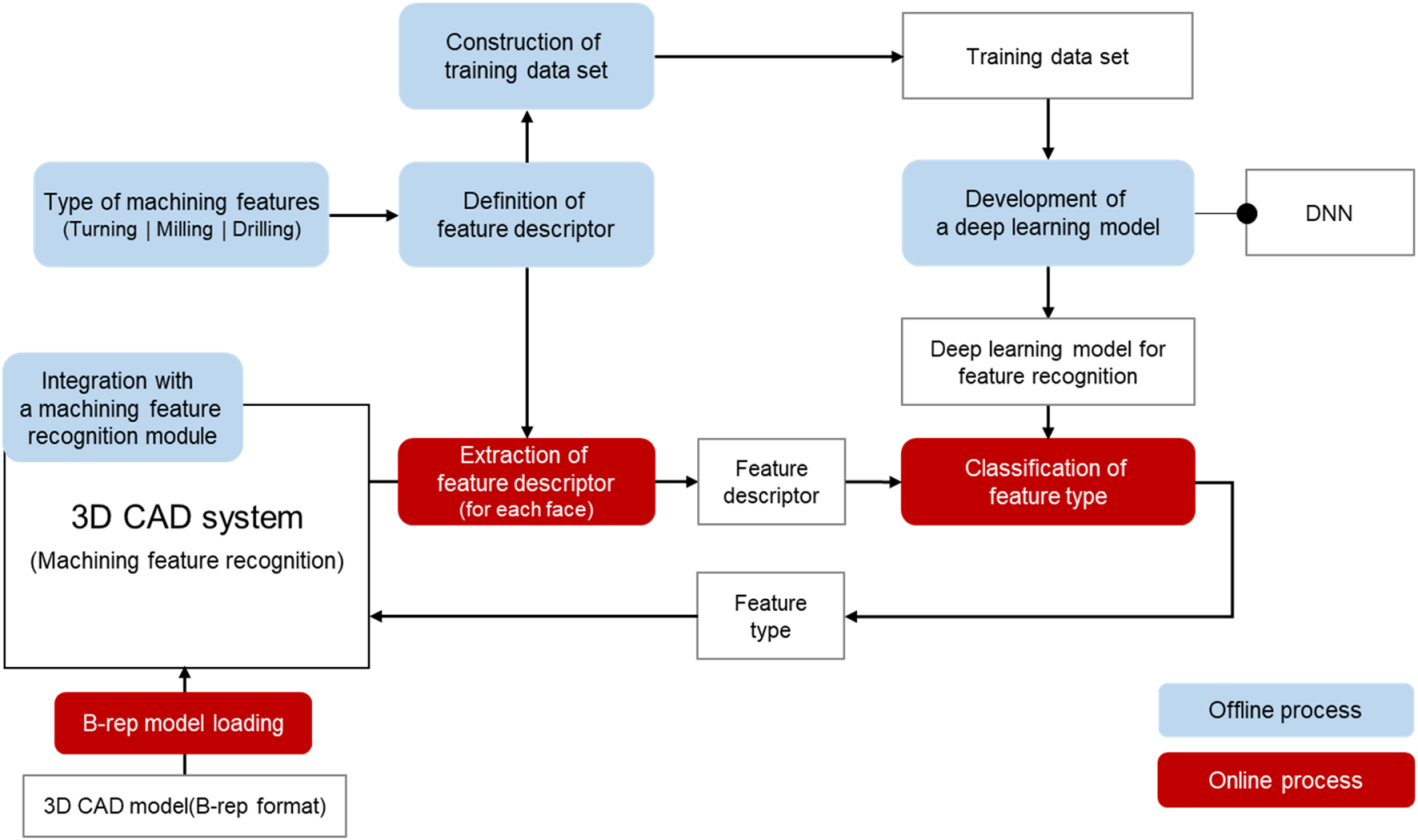
Proposed method for recognizing machining features.

The proposed method using deep learning-based machining feature recognition is shown in Fig. 3, comprising online and offline processes. The online process generates feature descriptors for each face of the B-rep model loaded into a 3D CAD system, inputs them into deep neural networks, and then classifies feature types of the face. Then, the recognized type is returned to the 3D CAD system. The offline process generates feature descriptors, builds a training dataset composed of them, and then trains the deep neural networks for feature recognition.

**Figure 4:**
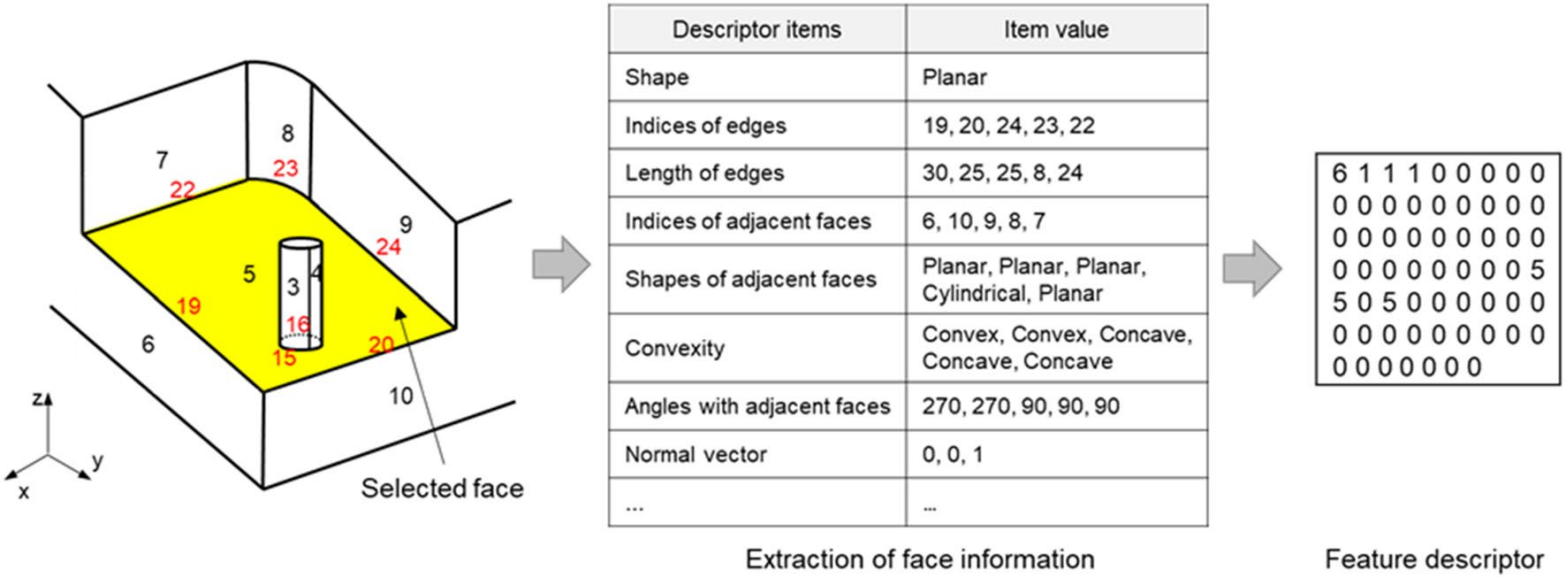
Generation of a feature descriptor from a 3D CAD system.

Previous feature recognition studies identified whether the associative pattern for the face and edge that make up the B-rep model is similar to a particular feature type pattern. Here, the associative patterns between many faces and edges are used for comparison. However, in this study, we defined a base face for each feature type and recognized the target face as the feature’s base face by identifying whether the target face’s attributes are similar to the particular feature’s base face. Here, as shown in Fig. 4, the feature descriptor explicitly represents and stores the main attributes of the face. Therefore, in this study, the machining feature recognition determines whether each face corresponds to a base face for each type of machining feature. Use of feature descriptors is effectively applicable even if interference between features makes it difficult or ambiguous to match faces or edges. Moreover, it is possible to extend the recognizable types of features by extending the descriptor.

The biggest challenge encountered when developing deep neural networks that directly use B-rep models as inputs to recognize machining features is the hierarchical complexity of B-rep models and the variability of data size. Thus, previous studies used neural networks after converting B-rep models into fixed-sized voxels or multiple images. Converted voxels or images with low resolution may not properly represent curves or surfaces. Furthermore, it may result in the loss of features (e.g., hole, pocket, fillet, or chamfer). Even if feature areas in a converted voxel or image are detected, the recognition of features in the B-rep model is challenging because of the resolution differences between the B-rep model and converted voxel or image. Additionally, the lack of training datasets about segmented 3D models for each area of feature makes it difficult to conduct relevant research.

**Figure 5:**
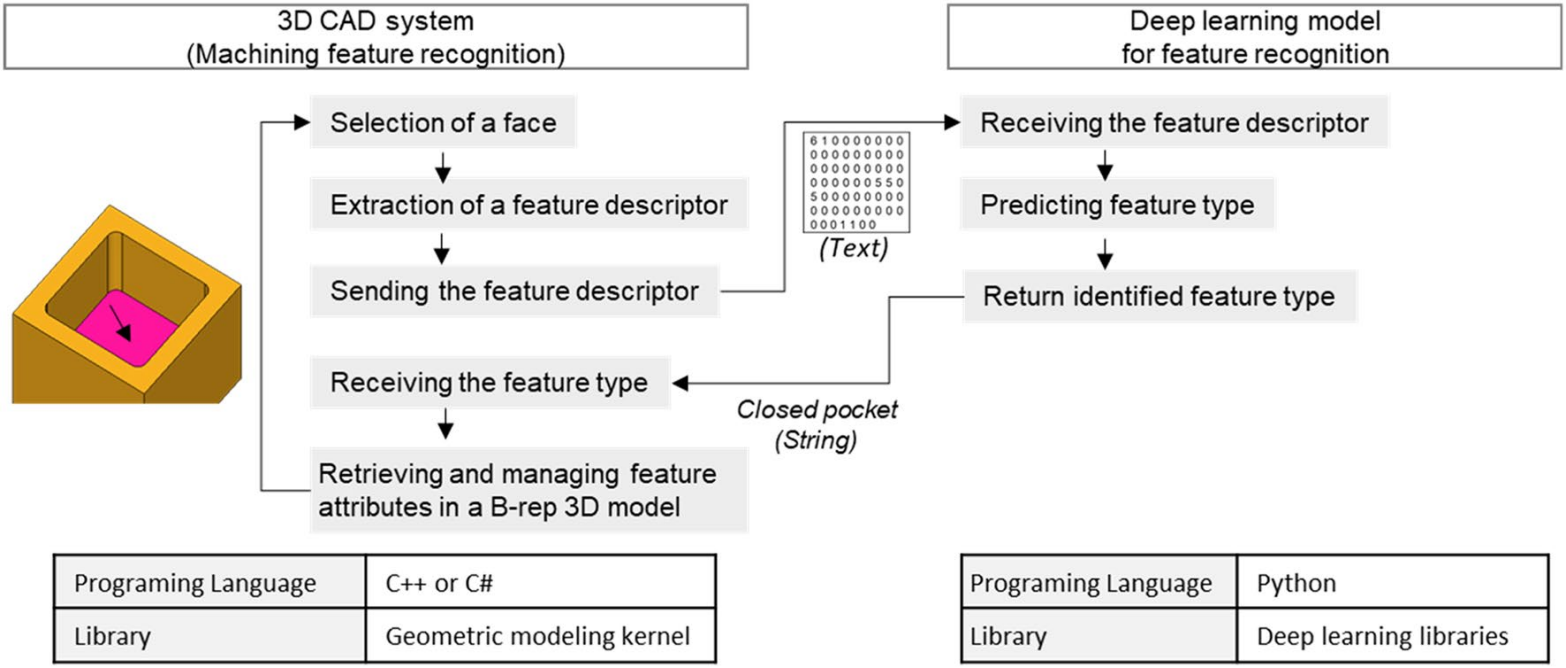
Tight integration between a 3D CAD system and deep learning model.

In the proposed method of machining feature recognition, input data of deep neural networks is the feature descriptor generated on each face of the B-rep model. Therefore, the proposed method is analogous to directly using the B-rep model information without conversion. Furthermore, this method can fix the input data format and size of deep neural networks because feature descriptors generated according to predefined structure for each face rather than those for a set of faces are used in feature recognition. The proposed method enables tight integration between a 3D CAD system and a deep learning model for machining feature recognition due to these characteristics, as shown in Fig. 5.

## Machining feature recognition using deep learning technology

### Feature descriptor

#### Base face of a machining feature

This study introduces the concept of a base face on machining features. The existing studies^3–26^ recognized most of the machining features from the relationship between the faces and the geometrical characteristics of each face. Referring to existing studies, we selected the base face for each feature in this study. Then, we devised a method to express the relationship between the faces constituting the feature and geometrical characteristics in terms of a descriptor based on this base face. The base face of a feature can be used as a reference for recognizing the features, even if the topology or geometry that makes up the feature partly changes. In other words, among the many faces that make up a feature, the base face best represents the feature characteristics. Figure 2 shows the base face for each type of feature covered in this study. For a hole, the base face depends on the number of faces that make up the hole. A simple hole or taper hole is a rotational shape, each with a cylinder and cone. A cylinder (cone) can be represented as either one cylindrical (conical) face or two half-cylindrical (half-conical) faces. This study assumes that a cylinder (cone) is represented by two half-cylindrical (half-conical) faces. The base face of a countersink hole or counterdrilled hole is the conical face. The base face of a counterbore hole is the planar face between the two cylindrical faces. For a slot, the base face, depending on the existence of the bottom face, is either the bottom or side face. For a pocket and an island, the base face is the bottom face of the pocket. For a fillet and chamfer, the cylindrical face or planar face is the base face.

#### Feature descriptor definition

The faces of the B-rep model have information about the faces themselves, information about the edges that make up the boundaries of the faces, information about the vertices that make up the edges, and relation information for the adjacent faces. The feature descriptor uses the type of face (e.g., planar face, cylindrical face, toroidal face, etc.), the normal vector, and loop type (inner or outer loop) for face information. Edges have the edge type (e.g., linear or curved type) and length. Vertices have coordinates information. Relation information for the adjacent face has the angle with the adjacent face, the convexity type, and the continuity type. Convexity is divided into concave or convex depending on the angle between the two adjacent faces. If the angle between the two adjacent faces is less than 180°, it is called concave, and if not, convex. Continuity is divided into C0, G1, C1, G2, and C2 according to the tangency and curvature between the two adjacent faces. Other continuities except C0 continuity have a tangent condition.

In a previous study^30^, we proposed feature descriptors to recognize machining features based on similarity comparison. The proposed feature descriptors, as shown in Table 1, include a type of base face, information on the relation of the base face with adjacent faces, parallel information of adjacent faces, and distance between parallel adjacent faces. Methods for using feature descriptors in the previous study^30^ are as follows. First, a feature descriptor *D*_*f*_ of the base face is defined for each feature type. Only the minimum information necessary for distinguishing features is stored in *D*_*f*_. Moreover, in the feature recognition phase, feature descriptor *D*_*i*_ is generated for each face *F*_*i*_ that makes up the B-rep model. The similarity of feature descriptor *D*_*i*_ is compared with *D*_*f*_ for each feature type. If the calculated similarity value is higher than the predefined threshold value, *F*_*i*_ is determined as the base face of the feature.

**Table 1:** The feature descriptor used in a previous study^30^.

Item description	Notation
Base face type	Face type
Number of adjacent faces in an outer loop	(Face type|convexity): number
Number of adjacent faces with G1 or higher continuity in an outer loop	(Face type|convexity): number
Number of perpendicular adjacent faces in an outer loop	(Face type|convexity): number
Number of adjacent faces in an inner loop	(Face type|convexity): number
Number of adjacent faces with G1 or higher continuity in an inner loop	(Face type|convexity): number
Number of perpendicular adjacent faces in an inner loop	(Face type|convexity): number
Number of pairs of parallel adjacent faces	Number
Distance between a pair of parallel adjacent faces	Length (If the number of pairs of parallel adjacent faces is two or more, the shortest distance is chosen.)

In this study, as shown in Table 2, a new feature descriptor suitable for applying deep learning technology was constructed by referring to the feature descriptor proposed in the previous study^30^. There are ten types of faces: Bezier, BSpline, Rectangular Trimmed, Conical, Cylindrical, Planar, Spherical, Toroidal, Linear Extrusion, and Revolution. If any face is not of analytic type, we mark it as Unknown. The curvature of the target face is represented as positive (if the target face is convex in the normal direction), negative (if the target face is concave in the normal direction), or flat (if the target face is flat).

**Table 2:** Definition of feature descriptor’s items for deep learning.

Feature descriptor’s item	Notation
Target face type	Face type
Curvature of target face	Curvature
Width of target face (for face-machining)	Result of comparison
Width of target face (for edge-machining)	Result of comparison
Adjacent faces in an outer loop	Face type|Convexity: number
Adjacent faces with C0 continuity in an outer loop	Face type|Convexity: number
Perpendicular adjacent faces in an outer loop	Face type|Convexity: number
Location and convexity of inner loops	Location|Convexity: number

The width of a target face has two types of information, face- and edge-machining types, which mark whether the target face is longer (marked as Longer) or shorter (marked as Shorter) based on the predefined threshold. The width of the target face is the distance between two adjacent planes that are parallel to each other. If there are no adjacent faces parallel to each other, the minimum distance is calculated between adjacent faces that are not in contact. In the face-machining type, the threshold means the maximum diameter used in machining. This item has been defined for identification between features of face-machining type (holes, slots, pockets, and islands). Among the features of face-machining types, only the width of the slot has a value smaller than the threshold^31^. If the width of the target face is less than the threshold, the target face is more likely to be determined to be a slot. In the edge-machining type, the threshold has been defined to identify the edge-machining type features (fillets and chamfers) from the face-machining type features. The threshold for edge-machining is inputted by the user, considering the following conditions. Since the width of the edge-machining is generally shorter than the width of the face-machining, the threshold of edge-machining should be smaller than the threshold of the face-machining. If the width of the target face is smaller than the threshold of the edge-machining type, the target face is more likely to be determined as an edge-machining type feature.

The adjacent face information in an outer loop shows the number of adjacent face type and convexity pairs for adjacent faces of the target face. Convexity is marked as Concave, Convex, and Unknown. If two faces have a tangent relation, the convexity is generally not considered. However, even in this case, convexity can be calculated using the cross product of the normal vectors on both the faces and direction vector of the edge shared by both sides^32^. If convexity is not calculated, it is marked as Unknown. Moreover, the adjacent face information in an outer loop also includes the number of adjacent face type and convexity pairs that have a C0 continuity relation and a perpendicular relation with the target face.

Finally, the number of location and convexity pairs represents the location of the inner loop in the target face and the convexity relation between the target face and inner loop. If the inner loop is a decomposition shape, such as a hole and pocket, it is marked as Convex. On the other hand, if the inner loop is a composition shape, such as an island, it is marked as Concave. If the inner loop’s center and the target face’s center are the same, it is marked as Center; otherwise, it is marked as Anywhere.

Table 3 shows the feature descriptor created in the base face of an Opened island according to the structure of the feature descriptor defined in this study. As shown in the table, the descriptor used in this study differs from those in the previous study^30^ in terms of the descriptor item. When feature descriptors are created, the values for all the items that make up the descriptor are recorded.

**Table 3:** Feature descriptor values of the Opened island.

Feature type	Feature descriptor’s item	Item’s value
Opened island	Target face type	Planar
	Curvature of target face	Flat
	Width of target face (for face-machining)	Longer
	Width of target face (for edge-machining)	Longer
	Adjacent faces in an outer loop	Planar|Concave: 3 Planar|Convex: 1 Cylindrical|Concave: 2
	Adjacent faces with C0 continuity in an outer loop	Planar|Concave: 3 Planar|Convex: 1 Cylindrical|Concave: 2
	Perpendicular adjacent faces in an outer loop	Planar|Concave: 3 Cylindrical|Concave: 2
	Location and convexity of inner loops	Anywhere|Concave: 2

### Deep neural network for feature recognition

#### Feature descriptor encoding

We used the integer encoding technique to apply feature descriptors in deep learning models. Integer encoding is a natural language processing technique, wherein the data format is converted from natural language to integer. In this section, we describe how to encode with the contents of Table 3 to aid understanding. We also describe the encoding method by dividing feature descriptor items into face, outer loop, and inner loop information.

For the type, curvature, and width items of the target face corresponding to the face information, different integer values were assigned according to the value of each descriptor item, as shown in Table 4. Table 5 shows an encoding example of the feature descriptor items regarding the target face information. As shown in the table, the descriptor’s items regarding a target face represent four integer values.

**Table 4:** Identifiers of descriptor item values regarding a target face.

ID	Item		
	Target face type	Curvature of target face	Width of target face (face-machining and edge-machining)
0	Unknown	Flat	–
1	Bezier	Positive	Longer
2	BSpline	Negative	Shorter
3	Rectangular trimmed	–	–
4	Conical	–	–
5	Cylindrical	–	–
6	Planar	–	–
7	Spherical	–	–
8	Toroidal	–	–
9	Linear Extrusion	–	–
10	Revolution	–	–

**Table 5:** Encoding of descriptor items regarding a target face.

Feature descriptor’s item	Item’s value	Encoding value
Target face type	Planar	6
Curvature of target face	Flat	0
Width of target face (for face-machining)	Longer	1
Width of target face (for edge-machining)	Longer	1

**Figure 6:**
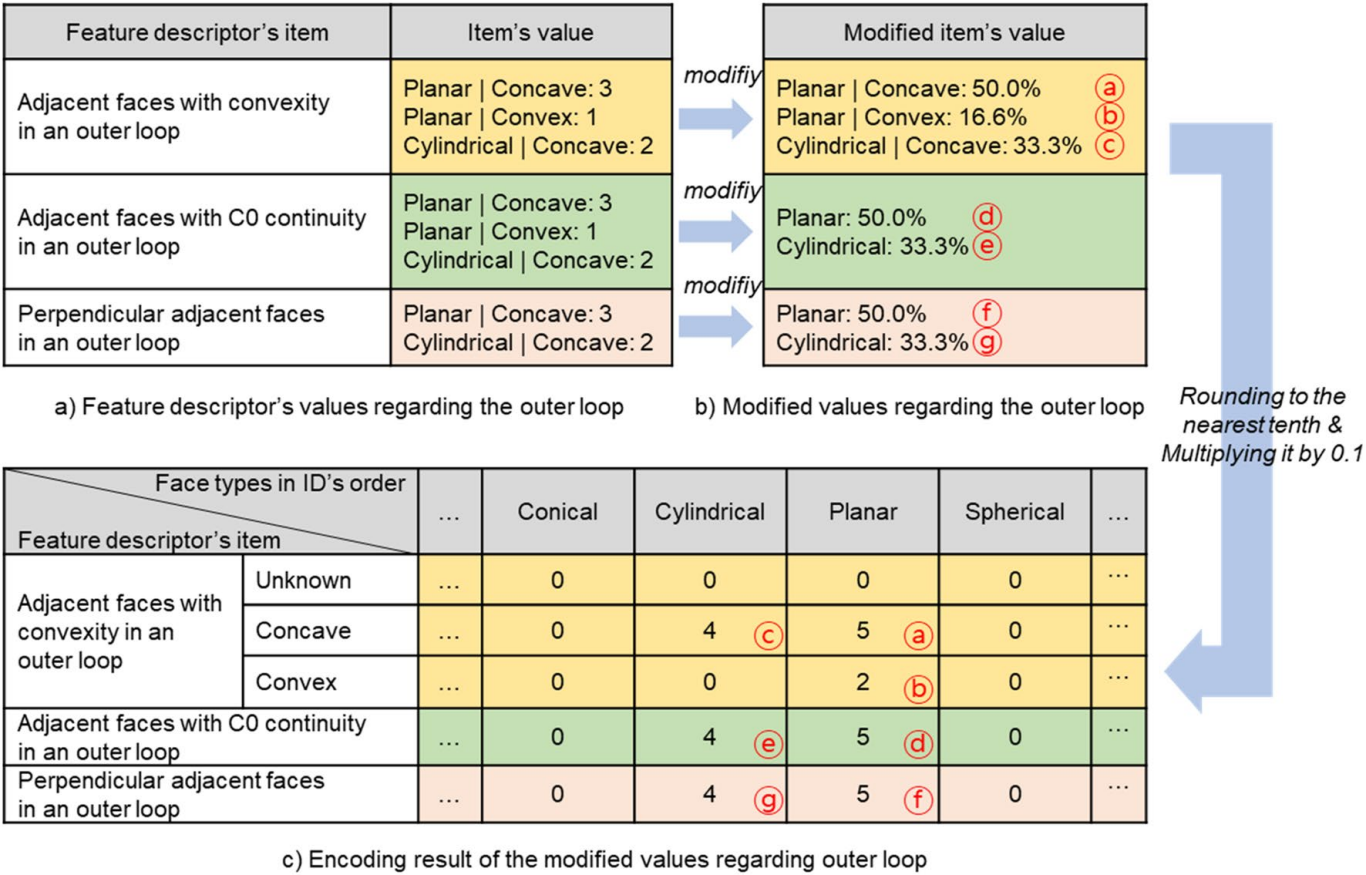
Encoding process of descriptor items regarding outer loop of a target face.

Outer loop information and descriptor item values are normalized in ratio form, as shown in Fig. 6, and then encoded as integers. The descriptor item values regarding the outer loop represent the number of adjacent faces corresponding to a specific type in the defined feature descriptor. Thus, the more adjacent faces, the larger the value of the descriptor’s item naturally attains. To prevent this issue, we have normalized the number of all adjacent faces that compose the outer loop by calculating the ratio of the adjacent faces of a specific type, as shown in Fig. 6b.

It is necessary to note here that the items of adjacent faces with C0 continuity in an outer loop and perpendicular adjacent faces in an outer loop calculate only the ratio of adjacent faces with concave convexity. If two faces in contact with each other are perpendicular, convexity must be concave. Additionally, if two faces in contact with each other are in a C0 continuity relation, convexity can be either convex or concave. However, most machining features have concave convexity because machining features are made into shapes by removing volumes from the stock. Thus, in these items, the ratio of adjacent faces of Concave convexity is calculated. An additional point to note is to calculate the ratio of the number of faces to the total number of adjacent faces. For example, for perpendicular adjacent faces in an outer loop shown in Fig. 6b, the ratio becomes 50% because the number of Planar face types is 3 and the total number of adjacent faces is 6.

When the normalization of the feature descriptor’s item values associated with outer loop information is completed, the normalized values are encoded, as shown in Fig. 6c. In the encoding process, the normalized value was rounded to the nearest tenth and then multiplied by 0.1 to ensure that the resulting value was represented as an integer value between 0 and 10. Encoding values were separately represented according to the type of faces (11 types) and the descriptor items (5 types) considering convexity, as shown in Fig. 6c. This process will result in 55 (11 × 5) descriptor values corresponding to the outer loop.

Features that utilize inner loop information in the machining feature classification include Islands and Counterbore holes. Counterbore holes typically have an inner loop in the form of “Center|Convex.” The Islands must have an inner loop in the form of “Anywhere|Concave.” Accordingly, the inner loop-related descriptor items were subdivided into “Anywhere|Concave” and “Center|Convex.” In the encoding process of an inner loop-related descriptor item, as shown in Table 6, if an inner loop corresponding to the above item was present, it was recorded as 1 and as 0 if it was not present. Table 6 shows two inner loops of “Anywhere|Concave” and no inner loops of “Center|Convex” inside a target face. Thus, it was recorded as 1 and 0.

**Table 6:** Encoding of descriptor items regarding inner loops of a target face.

Feature descriptor’s item	Item’s value	Encoding value
Location and convexity of inner loops	Anywhere|Concave: 2	1
	Center|Convex: 0	0

**Figure 7:**
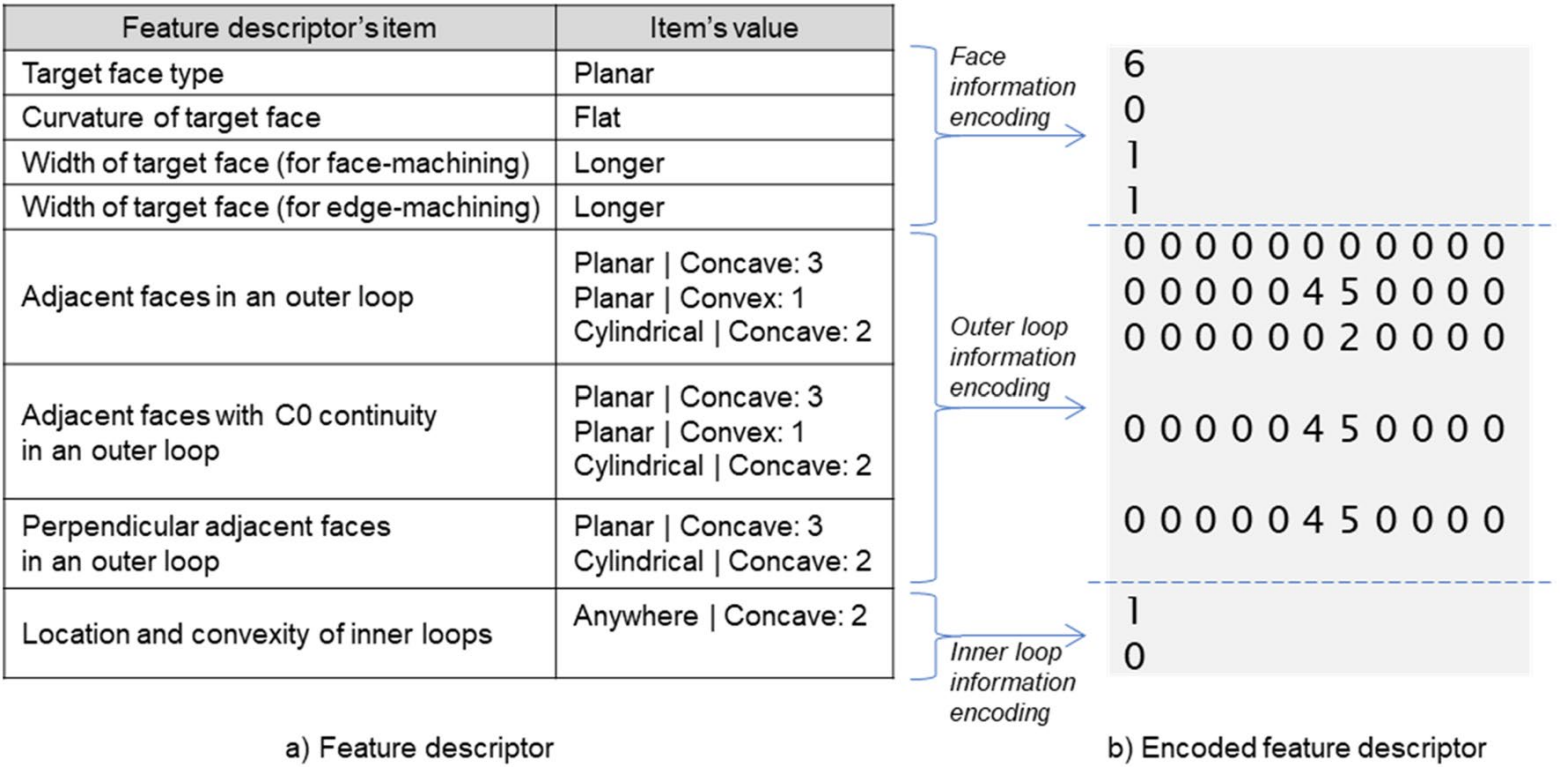
Integer encoding result of a feature descriptor.

After feature descriptor encoding, a feature descriptor (Fig. 7a) is encoded into a total of 61 integer arrays (Fig. 7b) to have 4 values in face information, 55 values in outer loop information, and 2 values in inner loop information. This feature descriptor has the same size regardless of the type of faces that makes up the B-rep model, making it easy to use as input for deep neural networks.

#### Development of the deep neural network for feature recognition

In this study, we have developed a deep neural network of standard feed-forward fully connected method for feature recognition. Deep neural network means an artificial neural network comprising one input layer, one output layer, and n hidden layers^33–36^.

**Figure 8:**
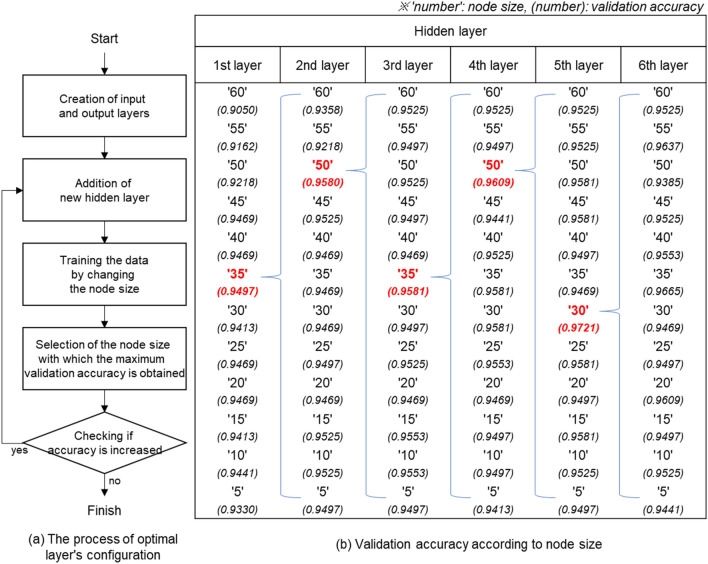
Experiment design to select an optimal configuration of a deep neural network.

To determine the optimal number of layers and node size of deep neural networks, training was performed by changing the number of hidden layers and node size according to the procedure given in Fig. 8a. We first fixed the node size of the input and output layers as 61 and 17, respectively. As the activation functions of the hidden and output layers, we used the ReLU and Softmax, respectively. As the loss function and optimization function of the neural network, we used Cross entropy loss and Adam optimizer, respectively. Then, we created the first hidden layer and trained while reducing the node size by an increment of 5 from 60, as shown in Fig. 8b, and selected the node size when the validation accuracy was the highest. When the number of nodes in the first hidden layer was determined, a new hidden layer was added to similarly select the optimal node size. With this procedure, we repeated the generation of hidden layers until the validation accuracy did not increase. Repeated experiments found that the validation accuracy no longer increased in the sixth hidden layer. Accordingly, the deep neural network herein comprises five hidden layers, with node sizes of 35, 50, 35, 50, and 30.

**Figure 9:**
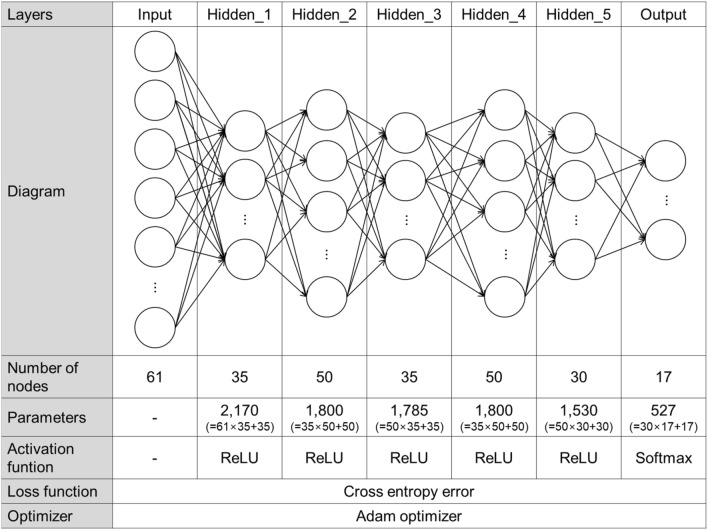
Configuration of the deep neural network for machining feature recognition.

To enhance the completion level of the deep neural network constructed in this study, we trained by applying dropout^37^ and batch normalization^38^ techniques. Table 7 shows the validation accuracy when the dropout layer and batch normalization layer are placed behind the nth hidden layer. As shown in Table 7, we can confirm that the validation accuracy is less when optimization techniques are applied than that when they are not applied. Based on Table 7, we confirmed that dropout and batch normalization techniques are not suitable for deep neural networks configured in this study. Through training experiments for the optimal configuration of neural networks, we finally developed the deep neural network configured as shown in Fig. 9.

**Table 7:** Validation accuracy when optimization techniques are applied to the deep neural network.

Techniques	Hidden layer					Validation accuracy
	1st	2nd	3rd	4th	5th	
Dropout	O	–	–	–	–	0.9469
	–	O	–	–	–	0.9525
	–	–	O	–	–	0.9609
	–	–	–	O	–	0.9553
	–	–	–	–	O	0.9497
Batch normalization	O	–	–	–	–	0.9441
	–	O	–	–	–	0.9525
	–	–	O	–	–	0.9358
	–	–	–	O	–	0.9469
	–	–	–	–	O	0.9358
None	–	–	–	–	–	0.9721

**Figure 10:**
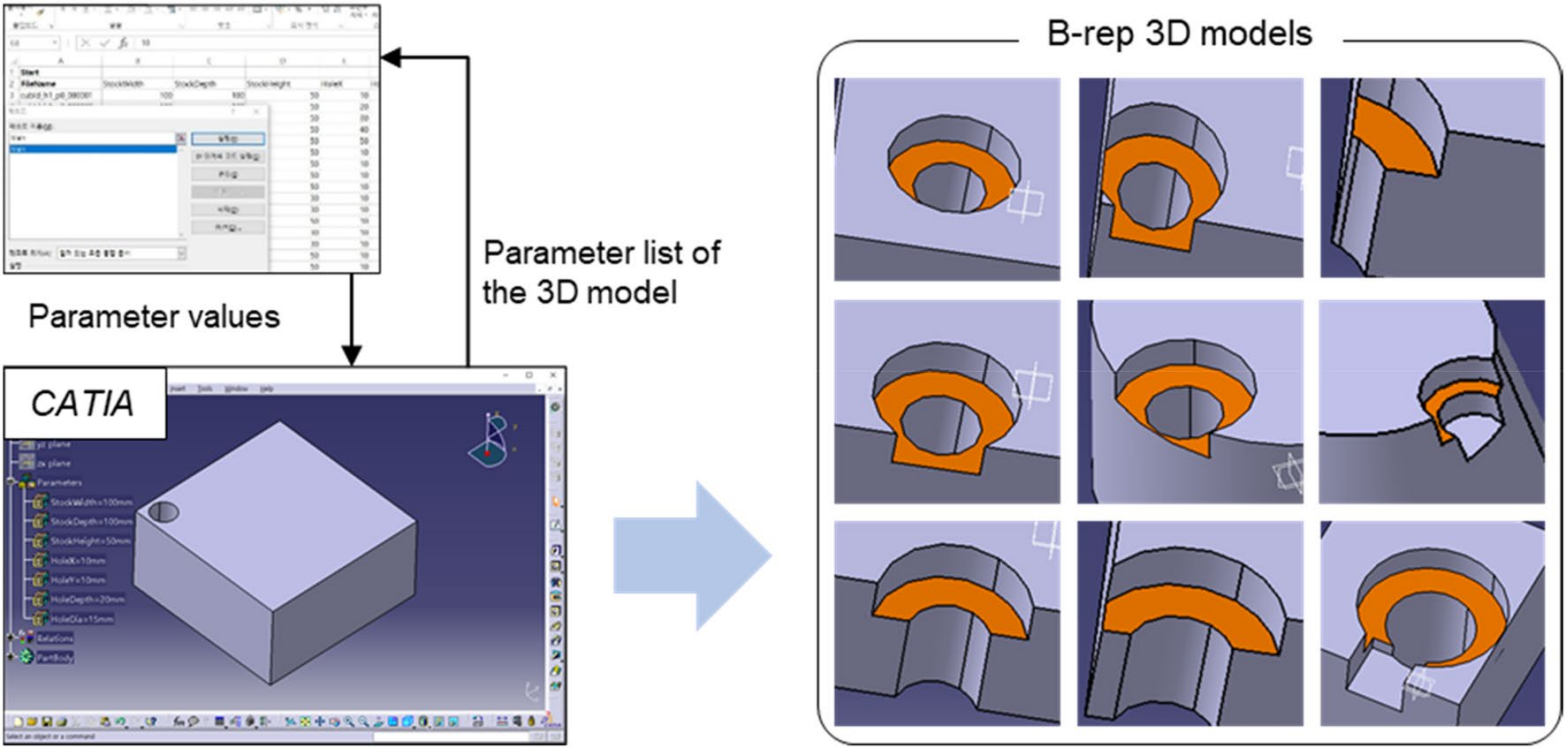
Parametric modeling to construct large numbers of B-rep models.

To construct training data for feature descriptors, we generated about 170,000 B-rep models through parametric modeling techniques using CATIA V5^39^ and Microsoft Excel^40^, as shown in Fig. 10. All B-rep models generated had one or more machining features, and the base face of each feature was given a different color. The reason for assigning different colors to the base face in the modeling process is to easily identify whether it corresponds to the base face having a feature on a particular face when creating a descriptor from the B-rep model. Since machining feature recognition aims to evaluate manufacturability, in this study, it is important to distinguish between machinable features and unmachinable features. Therefore, the generated dataset also contains unrealistic B-rep models.

**Figure 11:**
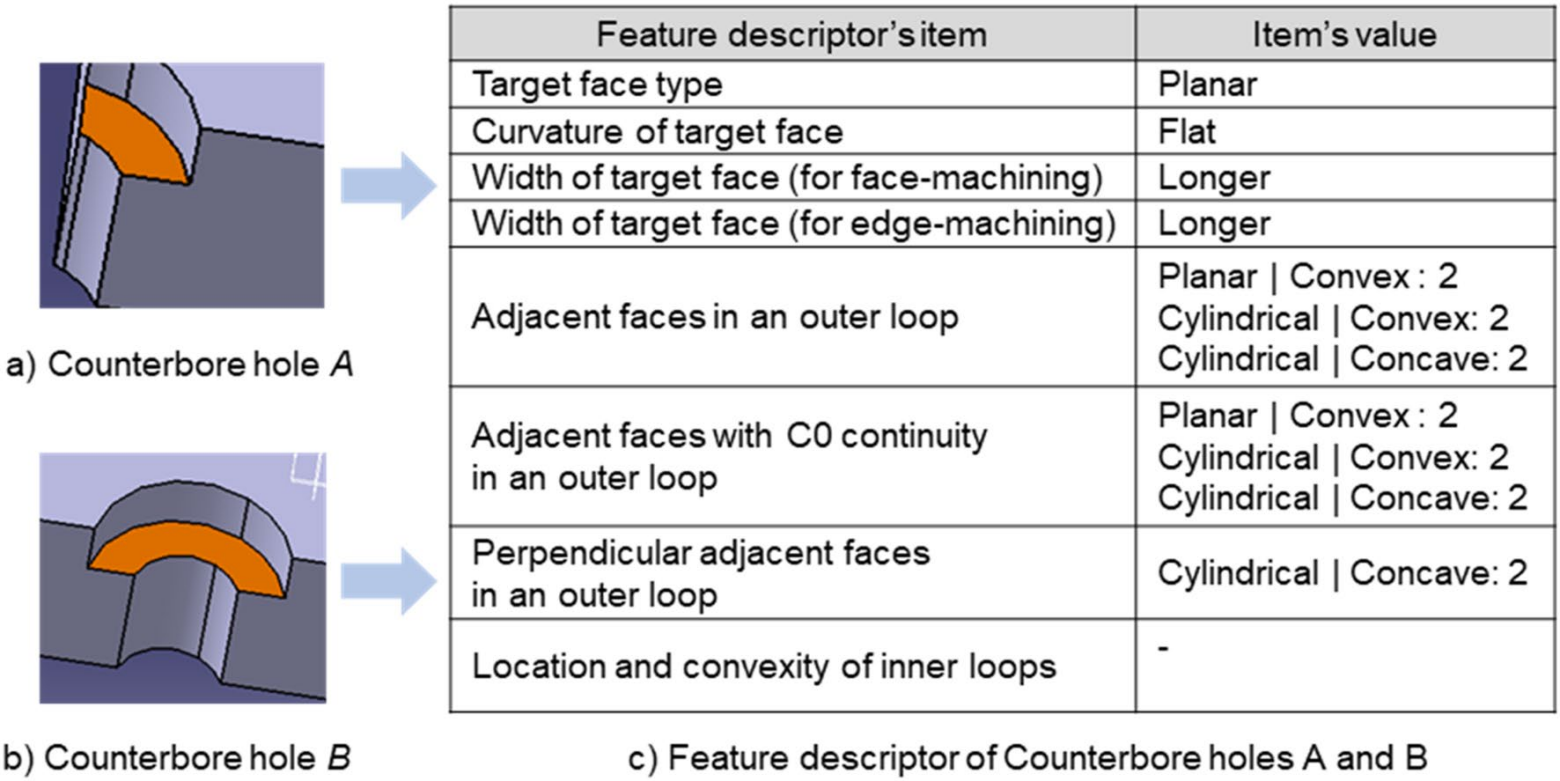
Different feature shapes having the same descriptor.

In the 170,000 B-rep models generated, many of them had different shapes but the same descriptors as shown in Fig. 11. We eliminated the duplicate data to prevent overfitting in the neural network’s training process.

**Figure 12:**
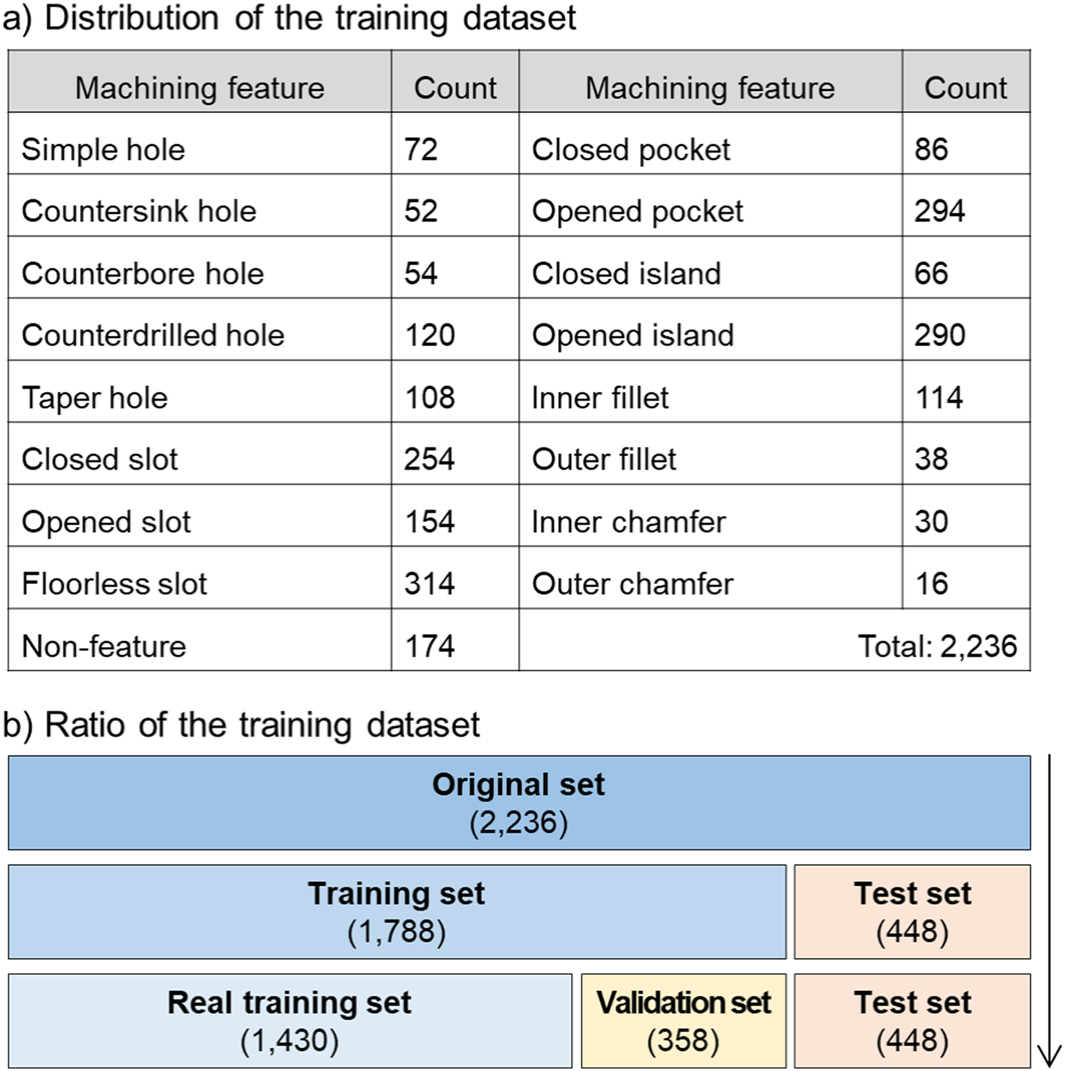
Overall information of the training dataset.

The training dataset for developing the deep neural network for machining feature recognition comprises 2236 feature descriptors, as shown in Fig. 12. This training dataset can be downloaded from the EIF lab homepage^41^. The composition of the training dataset is described in Fig. 12a. The training dataset contains feature descriptors for Simple hole (72), Countersink hole (52), Counterbore hole (54), Counterdrilled hole (120), Taper hole (108), Closed slot (254), Opened slot (154), Floorless slot (314), Closed pocket (86), Opened pocket (294), Closed island (66), Opened island (290), Inner fillet (114), Outer fillet (38), Inner chamfer (30), Outer chamfer (16), and Non-feature (174).

**Figure 13:**
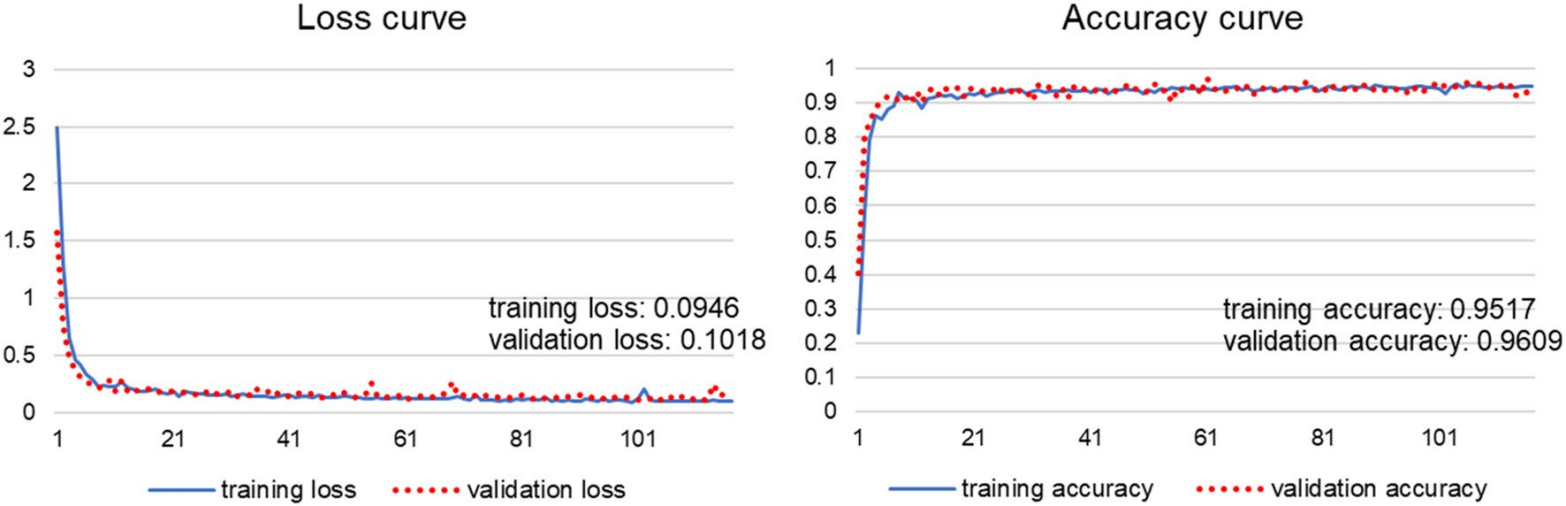
Learning curve of the developed deep neural network.

In the course of developing a deep neural network for the machining feature recognition, as shown in Fig. 12b, the entire dataset was divided by a ratio of 8:2, and 1788 feature descriptors were used as a training set. There is no set optimal division ratio between the training and test sets. Most of the existing studies randomly divide the training and test sets by a 7:3 or 8:2 ratio^42–45^. So, in this study, we randomly divided the entire dataset into training and test set by a ratio of 8:2. In the training set, the real training set and the validation set were randomly divided by a ratio of 8:2. As a result, the entire dataset was randomly divided into the real training set, validation set, and test set by a ratio of 64:16:20. For the training of deep neural networks, we set Batch size to 8 and Epoch to 1000. In the course of the training, deep neural networks showed a training accuracy of 0.9517, a training loss of 0.0946, a validation accuracy of 0.9609, and a validation loss of 0.1018, as shown in Fig. 13.

**Figure 14:**
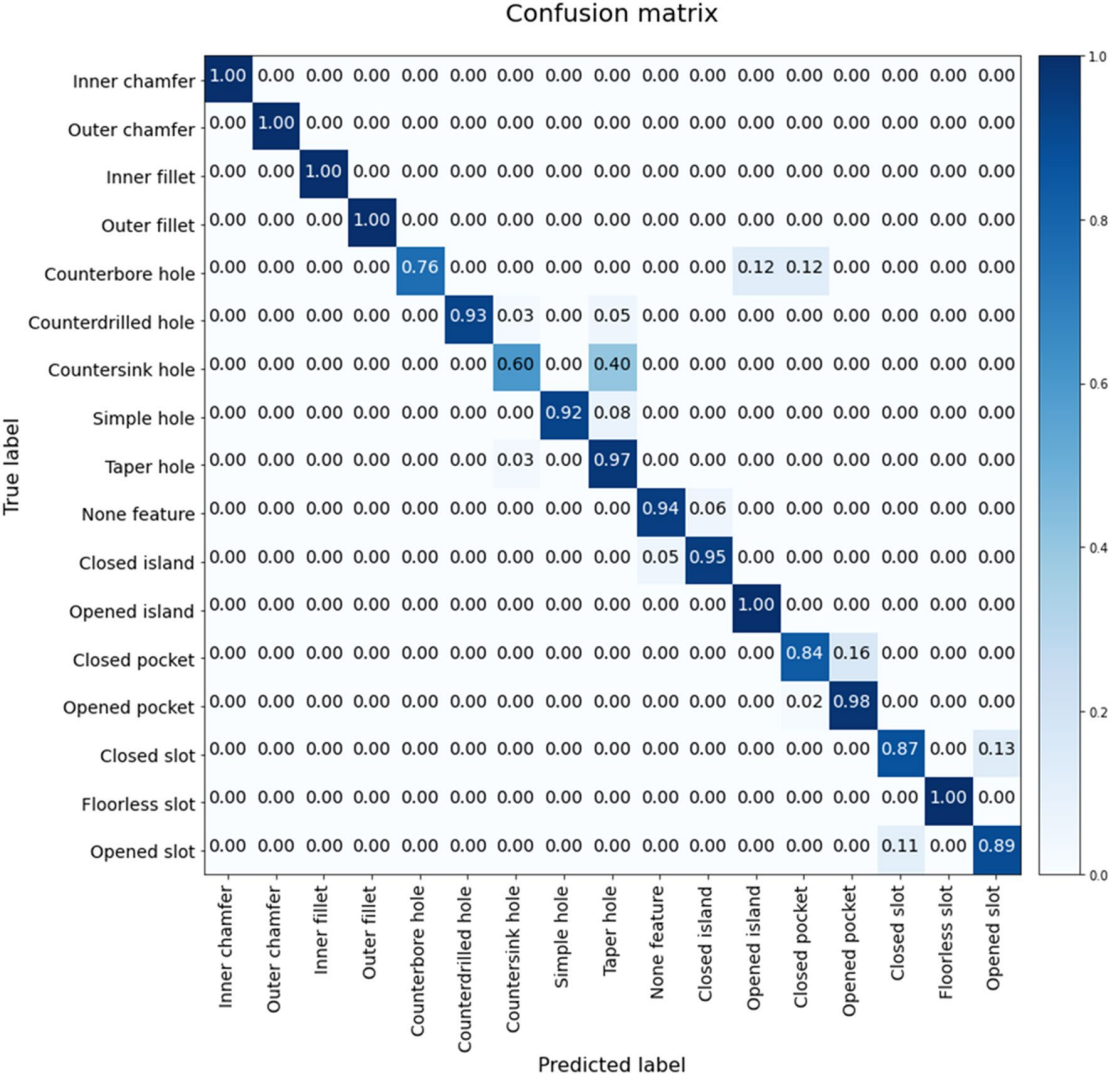
Confusion matrix of the developed deep neural network.

After the training of the deep neural networks, we validated the performance of the trained model with 448 feature descriptors that were not used in training. A confusion matrix was calculated, as shown in Fig. 14. The confusion matrix is primarily used to evaluate the performance of classification models and represents performance measures including accuracy, precision, and recall. As a result of validation, the trained deep neural network showed an accuracy of 0.9308, a mean precision of 0.9224, and a mean recall of 0.9108.

## Implementation and experimentation

### Implementation

**Figure 15:**
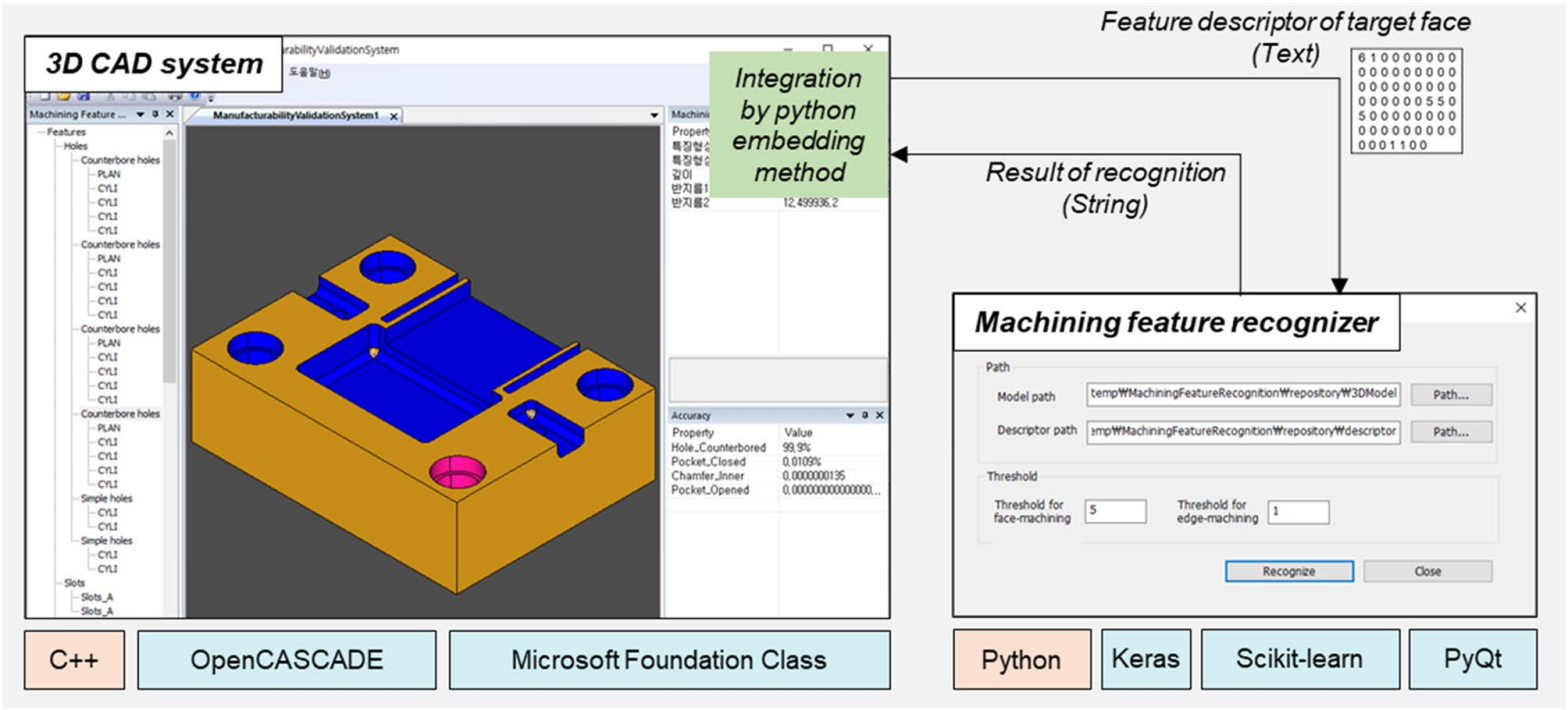
Prototype system development through integration of a 3D CAD system with machining feature recognition based on the deep neural network.

According to the proposed method for machining feature recognition, a prototype system was developed, as shown in Fig. 15. We implemented a module SW for feature recognition based on the deep neural network in Python language on the Windows 10 operating system. The PyQt library was utilized to configure the module’s GUI. The deep neural network was implemented using TensorFlow-based Keras and Scikit-learn libraries. In the 3D CAD system^30^ for feature recognition developed in the previous study, we integrated the developed recognition module SW using the python embedding method, as shown in Fig. 15. For module development and recognition experiments, computers with Intel Core i7 CPU, 64 GB RAM, and NVIDIA GeForce GTX 760 graphics card were used.

### Experimentation

The training dataset comprises descriptors that are generated from B-rep models by parametric modeling. These B-rep models have relatively simple shapes compared to the actual 3D models applied in the manufacturing field. Therefore, this experimentation tested the machining feature recognition of the complicated 3D models used in the field.

**Figure 16:**
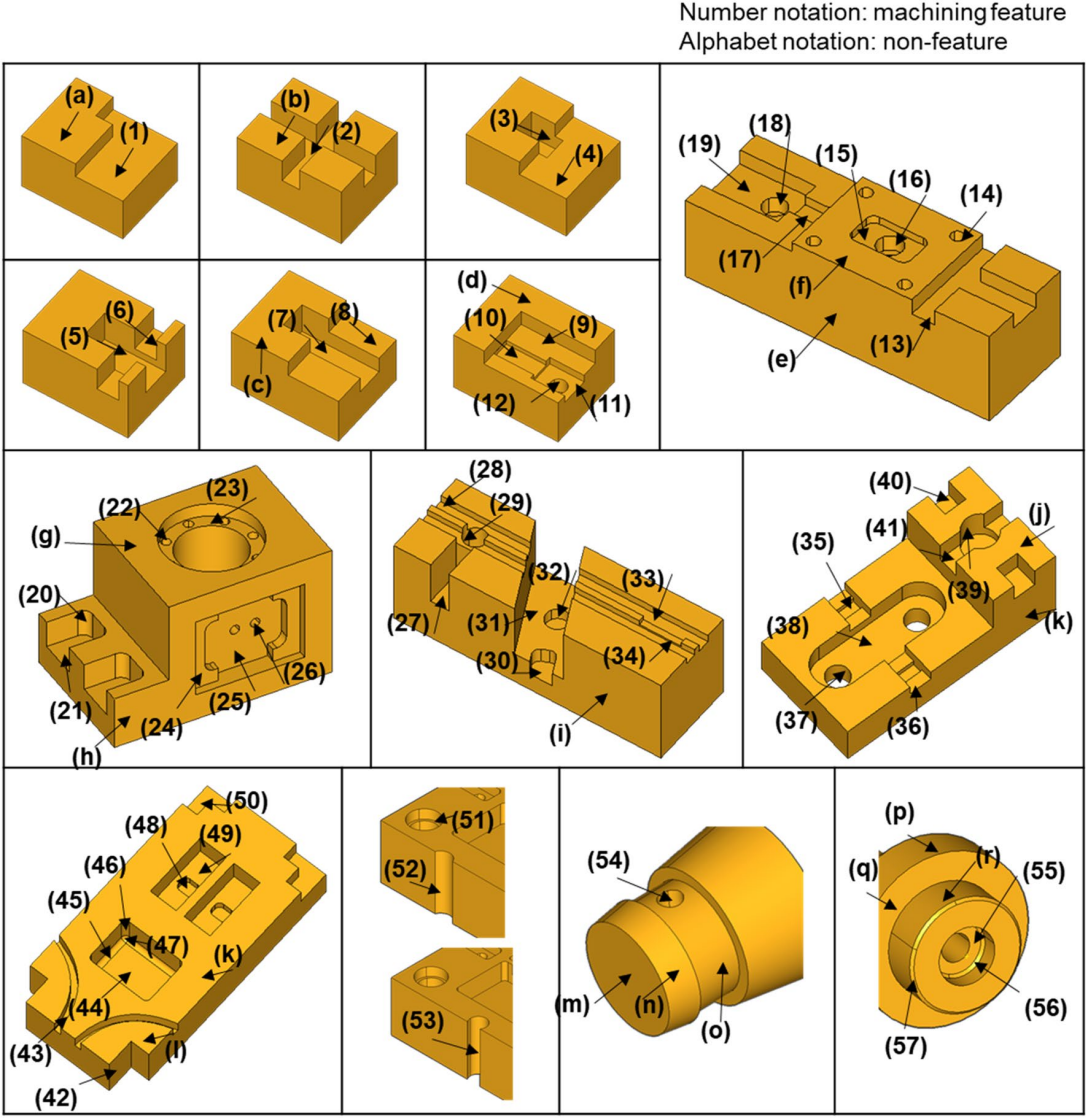
Test cases used in an experiment.

We prepared 15 B-rep models for the recognition experiment, as shown in Fig. 16, referring to the study^30,46–48^ on machining feature recognition. These 3D models are parts manufactured by turning, milling, and drilling, which possess 57 machining features and 18 non-features. Accordingly, the experiment was conducted for 75 test cases. Seventy-five test cases are the generated descriptors according to the procedure explained in “Feature descriptor encoding”. Nos. 15, 25, 38, and 52 of the test cases were recognition failures in the previous study^30^.

Table 8 shows the results of the recognition experiment of machining features on the test cases. The probability of columns 3 and 6 in Table 8 means the highest value among the possibility of feature labels. The possibility represents the likelihood that a particular feature label is correct. The results of the recognition experiment show that the feature type, which is true, was recognized as the first priority for all 75 test cases.

**Table 8:** Machining feature recognition result for the test case.

No.	Feature	Probability	No.	Feature	Probability
1	Opened pocket	99.9858	39	Simple hole	99.8326
2	Opened slot	99.1164	40	Opened pocket	99.9794
3	Closed pocket	78.1835	41	Opened pocket	99.9362
4	Opened pocket	77.4747	42	Opened pocket	77.4747
5	Opened pocket	99.9794	43	Opened slot	77.8519
6	Opened slot	99.4024	44	Closed pocket	97.2565
7	Opened pocket	99.9794	45	Inner fillet	100.0000
8	Opened pocket	77.4747	46	Inner fillet	100.0000
9	Opened pocket	98.8221	47	Inner fillet	99.5128
10	Opened pocket	99.9794	48	Closed pocket	99.4155
11	Opened pocket	99.9794	49	Closed pocket	78.1835
12	Simple hole	99.9977	50	Opened pocket	99.9893
13	Opened slot	99.3274	51	Counterbore hole	99.9996
14	Simple hole	99.9977	52	Simple hole	97.9496
15	Closed pocket	99.4155	53	Simple hole	99.9627
16	Counterbore hole	99.9996	54	Simple hole	99.9254
17	Opened pocket	99.9794	55	Counterbore hole	99.9526
18	Simple hole	99.9977	56	Inner chamfer	99.7046
19	Opened pocket	99.9794	57	Outer chamfer	99.2645
20	Inner fillet	100.0000	a	Non-feature	99.9615
21	Opened pocket	94.2895	b	Non-feature	99.9615
22	Simple hole	99.9977	c	Non-feature	99.9615
23	Counterbore hole	99.9996	d	Non-feature	99.9615
24	Opened pocket	99.9333	e	Non-feature	99.9615
25	Closed pocket	98.9804	f	Non-feature	99.9615
26	Simple hole	99.9977	g	Non-feature	99.9615
27	Opened pocket	99.9794	h	Non-feature	99.9615
28	Opened slot	95.4172	i	Non-feature	99.9615
29	Simple hole	99.9848	j	Non-feature	99.9188
30	Opened pocket	94.2895	k	Non-feature	99.9615
31	Opened pocket	99.0065	l	Non-feature	99.7054
32	Simple hole	99.9977	m	Non-feature	98.3473
33	Opened slot	99.4024	n	Non-feature	99.9921
34	Opened pocket	99.9858	o	Non-feature	94.0457
35	Closed pocket	78.1835	p	Non-feature	99.9995
36	Opened pocket	99.9858	q	Non-feature	99.9962
37	Simple hole	99.9627	r	Non-feature	99.9998
38	Closed pocket	99.4155			

Experimental results show that the deep neural network in this study calculated probabilities of over 90% on 68 of the 75 test cases. The remaining 7 test cases calculated the probability of the late 70% are Closed pockets (Nos. 3, 35, and 49), Open pockets (Nos. 4, 8, and 42), and Opened slots (No. 43). Nos. 3, 35, and 49 corresponding to Closed pockets have the same feature descriptor. Nos. 4, 8, and 42 corresponding to Opened pockets have the same feature descriptor.

To analyze features with probability of over 70%, we represented the descriptors of the two features (4 Opened pocket and 43 Opened slot), which exhibited the lowest probability, in the graph, as shown in Table 9. The graph’s horizontal axis represents the index of the feature descriptor encoded by an integer, and the vertical axis represents the value at each index. Column 1 of this table represents all descriptors corresponding to a particular type in the training data. Column 2 (column 3) represents a feature descriptor after the selection of a feature with probability of 90% or more (70% or more) from the experimental results.

**Table 9:**
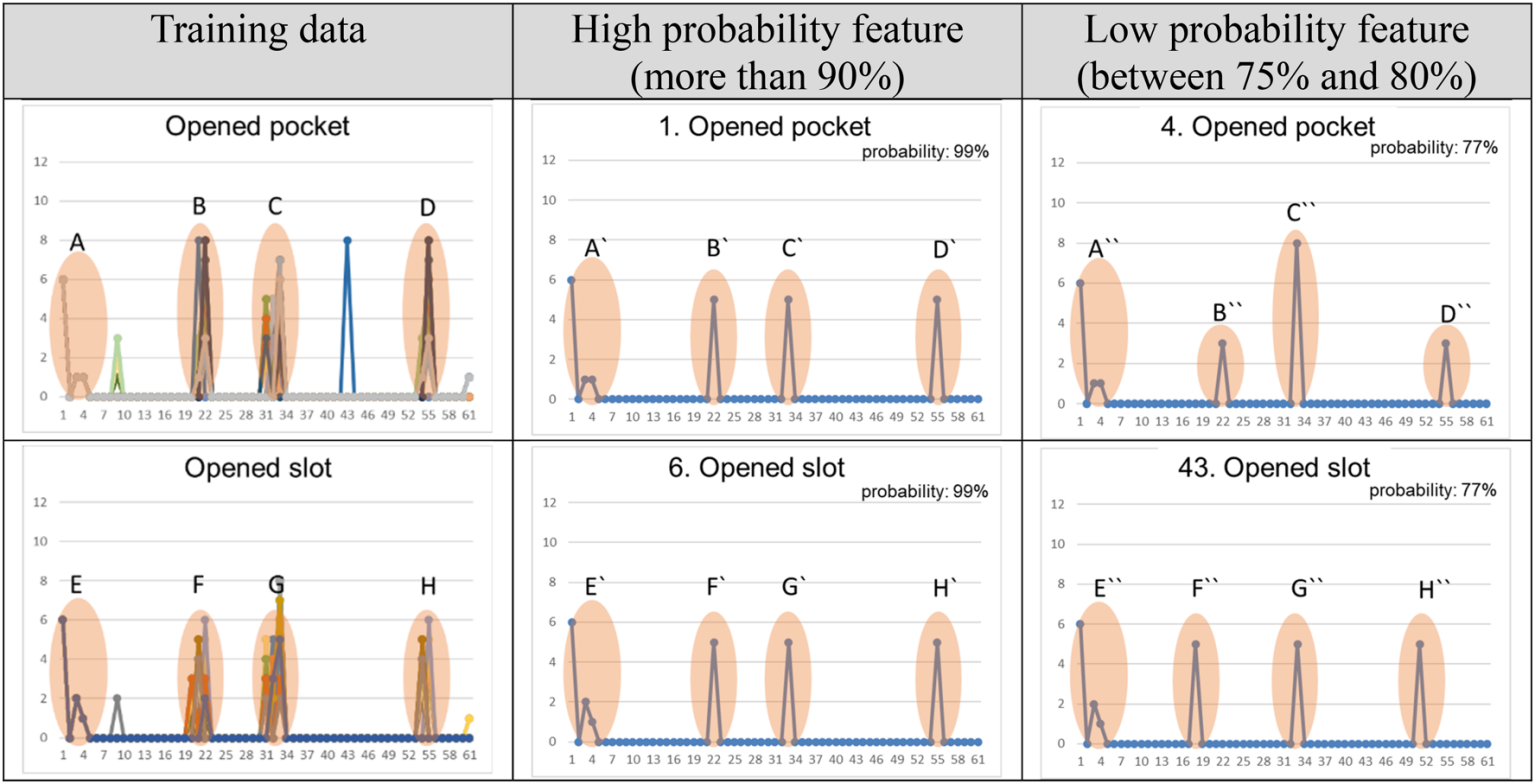
Analyses of machining features showing low probability.

The feature descriptor graphs in row 2 show that sections A, B, C, and D are important for determining the recognition target face as the Opened pocket. We can confirm that all graphs for the training data and the recognized faces completely correspond to each other in Section A. From the relative size of sections B, C, and D, the 1 Opened pocket tends to be more similar to the training data than the 4 Opened pocket. The graphs of the training data and the 1 Opened pocket are flat at sections B, C, and D, while the 4 Opened pocket has a relatively significant difference in the values of the sections. Consequently, the 4 Opened Pocket seemingly outputs a relatively low probability.

The feature descriptor graphs in row 3 show that sections E, F, G, and H are important for determining the recognition target face as the Opened slot. We can confirm that all graphs for the training data and the recognized faces completely correspond to each other in Section E. Sections F, G, and H show that the recognized face graphs tend to be similar to those in the training data. However, we can see that the values of sections F and H of the 43 Opened slot are located in different indexes. Consequently, the 43 Opened slot seems to output a relatively low probability, although it tends to be similar to that of the training data.

## Conclusions

We proposed a method of machining feature recognition based on the deep neural network using feature descriptors to ensure tight integration with 3D CAD systems. The proposed method supports 16 types of machining feature recognition. To recognize the machining features, the proposed method generates feature descriptors from the B-rep model’s face and recognizes feature types by inputting the descriptors into the deep neural network, and it returns the recognized feature types to the 3D CAD system.

We defined new feature descriptors suitable for the application of deep learning technology, referring to the descriptors proposed in a previous study^30^. Moreover, we used the integer encoding technique to apply the feature descriptor to the deep learning model. Since this technique can create feature descriptors having the same structure and size for each face composing the B-rep model, it can be easily used as input for the deep neural network.

The standard feed-forward fully connected method was applied to develop the deep neural network for machining feature recognition. The deep neural network has five hidden layers in addition to input and output layers. As the activation functions of the hidden and output layers, we used the ReLU and Softmax, respectively. In addition, as loss and optimization functions of the neural network, we used Cross entropy loss and Adam optimizer, respectively.

The training dataset used in the development of the deep neural network has a total of 2236 feature descriptors. We used 1788 training data for the learning of the deep neural network. We then tested the performance of the model with 448 feature descriptors that were not used for training. Consequently, the trained deep neural network showed an accuracy of 0.9308, a mean precision of 0.9224, and a mean recall of 0.9108.

In the experiment, we prepared 75 test cases for 15 B-rep models, referring to existing machining feature recognition studies^30,46–48^. In the recognition experiment for the test cases, 68 cases recognized true feature types with over 90% probability as the first priority, while the remaining seven cases recognized true feature types with over 70% probability as the first priority.

It is not easy to prove that the data for training is sufficient. So, for most DNN training, researchers generate as much different data as possible and ensure that the resulting data distribution is uniform. After creating 3D CAD models in this study, we generated the various data by transshaping the models through the parametric modeling method. The generated data distribution was made uniform by adjusting the case that transshapes for each 3D CAD model type. After training the proposed deep neural network with the generated dataset, we experimented with recognizing features with the proposed deep neural network for 75 test cases of Fig. 16. Through this experiment, we confirmed that the deep neural network trained well.

In the future, we will further subdivide the items that constitute the feature descriptor to expand recognizable machining feature types. We will also improve the probability of three feature types (Closed pockets, Opened pockets, and Opened slots) that show relatively low probability of over 70%. According to the subdivision of the items constituting the feature descriptors, we will increase the number of training data because the number of training data (2236) in this study may be insufficient. Finally, we plan to conduct a study to recognize machining features by applying a deep learning model, such as a convolutional neural network or a recurrent neural network, with higher performance than the deep neural network used in this study.

### Consent to participate

All authors consent for participation.

### Consent for publication

All authors consent for publication.

## Data Availability

Dataset used in this study is available from^41^.

## References

[1] Cheng K, Pan PY, Harrison DK (2001). Web-based design and manufacturing support systems: Implementation perspectives. Int. J. Comput. Integr. Manuf..

[2] Amrina, E., & Yusof, S. M. (2011). Key performance indicators for sustainable manufacturing evaluation in automotive companies. in *2011 IEEE international conference on industrial engineering and engineering management*, 1093–1097. IEEE.

[3] Joshi S, Chang TC (1988). Graph-based heuristics for recognition of machined features from a 3D solid model. Comput. Aided Des..

[4] Chuang SH, Henderson MR (1990). Three-dimensional shape pattern recognition using vertex classification and vertex-edge graphs. Comput. Aided Des..

[5] Gavankar P, Henderson MR (1990). Graph-based extraction of protrusions and depressions from boundary representations. Comput. Aided Des..

[6] Tang K, Woo TONY (1991). Algorithmic aspects of alternating sum of volumes. Part 1: Data structure and difference operation. Comput.-Aided Design..

[7] Kim YS (1992). Recognition of form features using convex decomposition. Comput. Aided Des..

[8] Sakurai H, Dave P (1996). Volume decomposition and feature recognition, Part II: Curved objects. Comput. Aided Des..

[9] Woo Y (2003). Fast cell-based decomposition and applications to solid modeling. Comput. Aided Des..

[10] Vandenbrande JH, Requicha AA (1993). Spatial reasoning for the automatic recognition of machinable features in solid models. IEEE Trans. Pattern Anal. Mach. Intell..

[11] Regli III, W. C. (1995). Geometric algorithms for recognition of features from solid models (Doctoral dissertation).

[12] Han J, Requicha AA (1998). Feature recognition from CAD models. IEEE Comput. Graph. Appl..

[13] Hong T, Lee K, Kim S (2006). Similarity comparison of mechanical parts to reuse existing designs. Comput. Aided Des..

[14] Ohbuchi, R., & Furuya, T. (2009). Scale-weighted dense bag of visual features for 3D model retrieval from a partial view 3D model. in *2009 IEEE 12th International Conference on Computer Vision Workshops, ICCV Workshops*, 63–70. IEEE.

[15] Liu YJ, Luo X, Joneja A, Ma CX, Fu XL, Song D (2013). User-adaptive sketch-based 3-D CAD model retrieval. IEEE Trans. Autom. Sci. Eng..

[16] Sánchez-Cruz H, Bribiesca E (2003). A method of optimum transformation of 3D objects used as a measure of shape dissimilarity. Image Vis. Comput..

[17] Prabhakar S, Henderson MR (1992). Automatic form-feature recognition using neural-network-based techniques on boundary representations of solid models. Comput. Aided Des..

[18] Onwubolu GC (1999). Manufacturing features recognition using backpropagation neural networks. J. Intell. Manuf..

[19] Öztürk N, Öztürk F (2001). Neural network based non-standard feature recognition to integrate CAD and CAM. Comput. Ind..

[20] Sunil VB, Pande SS (2009). Automatic recognition of machining features using artificial neural networks. Int. J. Adv. Manuf. Technol..

[21] Marchetta MG, Forradellas RQ (2010). An artificial intelligence planning approach to manufacturing feature recognition. Comput. Aided Des..

[22] Jian C, Li M, Qiu K, Zhang M (2018). An improved NBA-based STEP design intention feature recognition. Futur. Gener. Comput. Syst..

[23] Zhang Z, Jaiswal P, Rai R (2018). Featurenet: Machining feature recognition based on 3d convolution neural network. Comput. Aided Des..

[24] Shi P, Qi Q, Qin Y, Scott PJ, Jiang X (2020). A novel learning-based feature recognition method using multiple sectional view representation. J. Intell. Manuf..

[25] Peddireddy D, Fu X, Wang H, Joung BG, Aggarwal V, Sutherland JW, Jun MBG (2020). Deep learning based approach for identifying conventional machining processes from CAD data. Procedia Manufact..

[26] Zhang D, He F, Tu Z, Zou L, Chen Y (2020). Pointwise geometric and semantic learning network on 3D point clouds. Integr. Comput.-Aided Eng..

[27] Cheon SU, Mun D, Han S, Kim BC (2012). Name matching method using topology merging and splitting history for exchange of feature-based CAD models. J. Mech. Sci. Technol..

[28] Mun D, Han S (2005). Identification of topological entities and naming mapping for parametric CAD model exchanges. Int. J. CAD/CAM.

[29] Kim H, Yeo C, Lee ID, Mun D (2020). Deep-learning-based retrieval of piping component catalogs for plant 3D CAD model reconstruction. Comput. Ind..

[30] Yeo C, Cheon S, Mun D (2021). Manufacturability evaluation of parts using descriptor-based machining feature recognition. Int. J. Comput. Integr. Manufact. Online First..

[31] Abrahamsen M (2019). Spiral tool paths for high-speed machining of 2D pockets with or without islands. J. Comput. Design Eng..

[32] Asiabanpour B, Mokhtar A, Hayasi M, Kamrani A, Nasr EA (2009). An overview on five approaches for translating cad data into manufacturing information. J. Adv. Manuf. Syst..

[33] Chen, G., Parada, C., & Heigold, G. (2014). Small-footprint keyword spotting using deep neural networks. in *2014 IEEE International Conference on Acoustics, Speech and Signal Processing (ICASSP)*, 4087–4091. IEEE.

[34] Bengio Y, Courville A, Vincent P (2013). Representation learning: A review and new perspectives. IEEE Trans. Pattern Anal. Mach. Intell..

[35] Schmidhuber J (2015). Deep learning in neural networks: An overview. Neural Netw..

[36] Zhang D, Zou L, Zhou X, He F (2018). Integrating feature selection and feature extraction methods with deep learning to predict clinical outcome of breast cancer. IEEE Access.

[37] Baldi P, Sadowski PJ (2013). Understanding dropout. Adv. Neural. Inf. Process. Syst..

[38] Ioffe, S., & Szegedy, C. (2015). Batch normalization: Accelerating deep network training by reducing internal covariate shift. in *International conference on machine learning*, 448–456. PMLR.

[39] Dassault Systèmes. CATIA V5. https://www.3ds.com/. Accessed 1 March 2021

[40] Microsoft. Microsoft Excel. https://www.microsoft.com/. Accessed 1 March 2021

[41] EIF laboratory at KU. Training dataset for descriptor-based machining feature recognition. http://www.dhmun.net/home/Research_Data. Accessed 1 March 2021

[42] Havasi, M., Hernández-Lobato, J. M., & Murillo-Fuentes, J. J. (2018). Inference in deep gaussian processes using stochastic gradient hamiltonian monte carlo.

[43] Rácz A, Bajusz D, Héberger K (2021). Effect of dataset size and train/test split ratios in QSAR/QSPR multiclass classification. Molecules.

[44] Yadav, A., Jain, T., Verma, V. K., & Pal, V. (2021). Evaluation of machine learning algorithms for the detection of fake bank currency. in *2021 11th International Conference on Cloud Computing, Data Science & Engineering (Confluence)*, 810–815. IEEE.

[45] Basnet B, Chun H, Bang J (2020). An intelligent fault detection model for fault detection in photovoltaic systems. J. Sens..

[46] Ning F, Shi Y, Cai M, Xu W, Zhang X (2020). Manufacturing cost estimation based on the machining process and deep-learning method. J. Manuf. Syst..

[47] Han J, Pratt M, Regli WC (2000). Manufacturing feature recognition from solid models: a status report. IEEE Trans. Robot. Autom..

[48] Wang Q, Yu X (2014). Ontology based automatic feature recognition framework. Comput. Ind..

